# Socio‐demographic variation in diagnosis of and prescribing for common mental illnesses among children and young people during the COVID‐19 pandemic: time series analysis of primary care electronic health records

**DOI:** 10.1111/jcpp.14026

**Published:** 2024-06-15

**Authors:** Louise Jane Hussey, Evan Kontopantelis, Pearl L. H. Mok, Darren M. Ashcroft, Matthew J. Carr, Shruti Garg, Carolyn A. Chew‐Graham, Nav Kapur, Karina Lovell, Roger T. Webb

**Affiliations:** ^1^ Division of Psychology and Mental Health, Manchester Academic Health Sciences Centre The University of Manchester Manchester UK; ^2^ Division of Informatics, Imaging and Data Sciences University of Manchester Manchester UK; ^3^ Division of Pharmacy and Optometry, School of Health Sciences, Manchester Academic Health Sciences Centre The University of Manchester Manchester UK; ^4^ Division of Pharmacy and Optometry, Centre for Pharmacoepidemiology and Drug Safety, Manchester Academic Health Sciences Centre The University of Manchester Manchester UK; ^5^ Division of Psychology and Mental Health, Faculty of Biology, Medicine and Health University of Manchester Manchester UK; ^6^ School of Medicine, Faculty of Medicine and Health Sciences Keele University Keele UK; ^7^ Division of Nursing, Midwifery & Social Work, University of Manchester Greater Manchester Mental Health NHS Foundation Trust Manchester UK

**Keywords:** Anxiety disorders, depression, COVID‐19, children and young people, ethnicity, deprivation, general practice

## Abstract

**Background:**

The impact of the COVID‐19 pandemic on the mental health of children and young people (CYP) has been widely reported. Primary care electronic health records were utilised to examine trends in the diagnosing, recording and treating of these common mental disorders by ethnicity and social deprivation in Greater Manchester, England.

**Methods:**

Time‐series analyses conducted using Greater Manchester Care Record (GMCR) data examined all diagnosed episodes of anxiety disorders and depression and prescribing of anxiolytics and antidepressants among patients aged 6–24 years. The 41‐month observation period was split into three epochs: Pre‐pandemic (1/2019–2/2020); Pandemic Phase 1 (3/2020–6/2021); Pandemic Phase 2 (7/2021–5/2022). Rate ratios for all CYP specific to sex, age, ethnicity, and neighbourhood‐level Indices of Multiple Deprivation (IMD) quintile were modelled using negative binomial regression.

**Results:**

Depression and anxiety disorder rates were highest in females, CYP aged 19–24, and White and ‘Other’ ethnic groups. During Pandemic Phase 1, rates for these diagnoses fell in all demographic subgroups and then rose to similar levels as those recorded pre‐pandemic. In Pandemic Phase 2, rates in Black and Mixed‐ethnicity females rose to a significantly greater degree (by 54% and 62%, respectively) than those in White females. Prescribing rates increased throughout the study period, with significantly greater rises observed in non‐White females and males. The temporal trends were mostly homogeneous across deprivation quintiles.

**Conclusion:**

The observed fluctuations in frequency of recorded common mental illness diagnoses likely reflect service accessibility and patients' differential propensities to consult as well as changing levels of distress and psychopathology in the population. However, psychotropic medication prescribing increased throughout the observation period, possibly indicating a sustained decline in mental health among CYP, and also clinicians' responses to problems presented. The comparatively greater increases in frequencies of diagnosis recording and medication prescribing among ethnic minority groups warrants further investigation.

## Introduction

Globally, mental health problems affect 10%–20% of children and young people (CYP), accounting for a large portion of the worldwide disease burden. The impact of these conditions reaches beyond childhood, with a substantial proportion of mental health problems in adults originating from mental well‐being experienced as a child and adolescent (Kieling et al., 2011). Prevalence is also reported to have increased in recent years. The latest follow‐up report to the 2017 Mental Health of Children and Young Persons in England Survey (Newlove‐Delgado et al., 2022) found that 18% of children aged 7–16 years had a probable mental disorder, an increase from 12% in 2017.

The impact of the coronavirus (COVID‐19) pandemic on the mental well‐being of CYP has been widely reported (Kauhanen et al., 2023; Samji et al., 2022; Trafford et al., 2023). A high prevalence of COVID‐19‐related fear was noted among children and adolescents, as well as more depressive and anxious symptoms compared with pre‐pandemic estimates (Kauhanen et al., 2023). In response to rising infection rates, the UK Government mandated a national lockdown on 23rd March 2020 (Brown & Kirk‐Wade, 2021). As such, in addition to the psychological stressors attributed to fear of the virus, other factors such as social isolation, parental stress, undetected child abuse, social media and online bullying worsened the impact of the pandemic (Kauhanen et al., 2023). Several UK studies found decreased mental well‐being in CYP when comparing pre‐pandemic rates with time periods during the pandemic (Banks & Xu, 2020; Bignardi et al., 2021; Gray et al., 2020; Niedzwiedz et al., 2021; Pierce et al., 2020). These investigations have all been based on self‐reports with individuals completing validated and standardised measures of mental health and well‐being.

Other studies examining the impact of the pandemic on changes in mental health in Greater Manchester (GM) and the wider UK population have used data from primary care electronic health records (Carr et al., 2021; Steeg et al., 2021; Williams et al., 2020). These studies revealed a marked decrease in recorded self‐harm episodes and mental illness diagnoses in patients aged 10–17 years during the early months of the pandemic. Reduced frequency of primary care consultation could be a consequence of patients' reluctance to attend healthcare facilities due to fears about exposure to the virus, as well as a concern to not overburden National Health Service (NHS) resources with non‐COVID conditions (Williams et al., 2020). When comparing the total number of GP appointments in England from April to August 2020 to the same period in 2019, there was a decrease of 20.8%, from 120.66 million to 95.52 million (NHS Digital, 2020a). There were also significant changes to the delivery of healthcare, with a reduction in face‐to‐face contact with GP consultations being predominantly carried out remotely. Pre‐pandemic, over 70% of consultations were carried out face‐to‐face; this had fallen to 23% once lockdown measures had been introduced (Marshall, Howe, Howsam, Mulholland, & Leach, 2020).

GM is a large conurbation with approximately 2.8 million residents in the North‐West region of England. It is more ethnically diverse than the whole population of England and has relatively high levels of deprivation. Around a third of the conurbation's population live among the fifth most deprived areas in England (Visit North West, 2023). As such, pressure on local healthcare systems is great, with life expectancy in eight of the ten metropolitan boroughs in GM being lower than the national average (Codling & Allen, 2020). It has long been established that there is an increased risk of mental health conditions for persons of lower socioeconomic position (Kivimäki et al., 2020) and among ethnic minorities (Smith et al., 2020). Evidence has shown that during the pandemic, deprivation and differential access to healthcare services widened these existing inequalities (Lee & Singh, 2021; Smith, Bhui, & Cipriani, 2020).

There were many different stages in the UK's COVID‐19 response, with the easing of restrictions followed by reimposition in response to rising infection rates (Brown & Kirk‐Wade, 2021). GM experienced some of the most stringent measures over lengthier time periods. There were also local lockdowns for areas with the highest infection rates (including the GM boroughs Bolton and Oldham), and when the ‘Tier System’ was introduced in September 2020, the entire GM conurbation was placed in Tier Three, the most restrictive category. Non‐pharmaceutical public health measures were lifted across the whole country in July 2021. Evidence has shown how there was a decrease in recorded episodes of mental health conditions during the early stages of the pandemic (Carr et al., 2021; Steeg et al., 2021; Williams et al., 2020). However, it is not yet known how rates of GP‐diagnosed mental illness changed after societal restrictions were lifted.

In this study, we utilised primary care electronic health records across GM to examine how rates of common mental illness diagnosis (anxiety disorders and depression) in CYP changed during the acute phase of the pandemic and since societal restrictions were lifted compared to pre‐pandemic levels. In addition, we assessed whether there was any change in the prescribing rates of associated psychotropic medication (anxiolytics and antidepressants). We also aimed to investigate whether these outcome frequencies varied by sex, age, ethnicity and neighbourhood‐level Index of Multiple Deprivation (IMD).

## Methods

### Data sources, study design and data access approval

We conducted a retrospective time‐series analysis using data extracted from the Greater Manchester Care Record (GMCR). This resource (set up in 2019) holds information from primary care electronic health records across GM. The dataset contains approximately three million patient records from 443 GP practices across all 10 GM metropolitan boroughs (Health Innovation Manchester, 2023). Anonymised data became available for research purposes as part of the COVID‐19 response. To enable access, a research protocol had to be approved in accordance with the national Control of Patient Information (COPI) notice (NHS Digital, 2020b).

### Data criteria and preparation

We requested data for monthly totals of all relevant clinical codes related to episodes of anxiety disorders and depression among patients aged 6–24 years from 1 January 2019 (first date available) to the latest available data available (31 May 2022) at the time of request (9 June 2022). In addition, we obtained information on all anxiolytic and antidepressant medications prescribed. Anxiolytics included medications classified as: barbiturates, benzodiazepines, non‐benzodiazepine benzodiazepine receptor agonists (NBBRAs) and ‘other’ anxiolytics or hypnotics. Antidepressants included monoamine oxidase inhibitors (MAOIs), norepinephrine reuptake inhibitors (NRIs), serotonin antagonist and reuptake inhibitors (SARIs), serotonin modulator and stimulator (SMS), serotonin and norepinephrine reuptake inhibitors (SNRIs), selective serotonin reuptake inhibitors (SSRIs), tricyclic antidepressants, tetracyclic antidepressants and ‘other’ antidepressants. Clinical code lists were provided by GMCR research staff (optimum clinical code list established previously for other study requests) and medication lists were provided by one of the co‐authors (MC, a senior academic pharmacist). Across the study's 41‐month observation period, these counts were stratified by sex, age, ethnicity and neighbourhood‐level IMD quintile 2019. The IMD is a measure of deprivation at a small area level across England, based on seven domains: income, employment, education, health, crime, housing and living environment (Department for Levelling Up, 2020). To calculate rates, we generated monthly estimates of GP population denominators. This was calculated using a triangulation of three data sources (GMCR, Census 2021 and NHS Digital GP practice lists). A month‐by‐month population denominator was not available from the GMCR. However, we were provided with a ‘snapshot’ of 1 month (December 2021). Monthly GP practice size information was provided by NHS Digital (NHS Digital, 2023). The demographic distribution was validated against 2021 Census (Office for National Statistics, 2023) information, and the proportional distribution by sex, age, ethnicity and IMD was applied to the GM population.

Rates during the pandemic were compared with the preceding pre‐pandemic period by dividing the 41‐month observation period (January 2019 to May 2022) into the following three time periods:

**Table:** 

Pre‐pandemic	Jan 2019‐Feb 2020 (months 1–14)
Pandemic Phase 1 (societal restrictions in place)	March 2020–June 2021 (months 15–30)
Pandemic Phase 2 (all restrictions lifted)	July 2021–May 2022 (months 31–41)

### Statistical analyses

The number of episodes of anxiety disorder and the number of episodes of depression were aggregated to give monthly totals and then subsequently grouped into the three time periods described above. Anxiolytic and antidepressant prescription counts were combined, and the monthly totals of prescriptions issued associated with an episode of anxiety disorder or an episode of depression were calculated. This gave us four study outcomes:

- The number of episodes of anxiety disorders
- The number of episodes of depression
- The number of anxiolytic/antidepressant prescriptions issued for anxiety disorder episodes.
- The number of anxiolytic/antidepressant prescriptions issued for episodes of depression.

Monthly episode/prescribing rates and rate ratios (and their 95% confidence intervals) were estimated using negative binomial regression, adjusted for seasonality by including calendar month (1–12) in the model. Rate ratios compared rates during Pandemic Phases 1 and 2 with the pre‐pandemic period, with the latter being the reference category. In addition, rate ratios specific to socio‐demographic groups (by sex, age group, ethnicity, IMD quintile) were calculated by fitting an interaction term between the demographic group of interest and the time period. All analyses were performed in Stata v17.

## Results

### Episode rates across the whole study period

Across the 41‐month observation period, the study dataset held monthly counts of 136,953 anxiety disorders and 132,101 depression episodes (Table 1). The volumes of anxiolytics/antidepressants prescribed for episodes of anxiety disorders (505,408) and depression (499,447) were similar. Episode and prescribing rates in females were approximately double the rates observed in males. Rates were much higher for the 19–24 age group than they were for those aged 6–18. For ethnicity, rates were highest for individuals described as White and for ‘Other’ ethnicities and were consistently lower for all outcomes among individuals categorised within the Black ethnic group. Rates were generally found to be lower in less deprived neighbourhoods. However, this pattern was not observed for patients within the most deprived quintile (IMD1). Rates for IMD1 were mostly found to be lower than those residing in neighbourhoods categorised within the second most deprived quintile (IMD2). Table S1 shows the rate ratios comparing these various sociodemographic groups.

**Table 1:** Population denominators, numerator values and diagnosed episode and prescribing rates of anxiolytics and antidepressants per 100,000 persons for anxiety disorders and depression

	Population denominators *N* (%)	Episodes				Medication prescribed			
		Anxiety disorders		Depression		Anxiety disorders		Depression	
		*n*	Rate per 100,000 persons	*n*	Rate per 100,000 persons	*n*	Rate per 100,000 persons	*n*	Rate per 100,000 persons
Gender									
Males	379,679 (50.8)	42,925	276	43,631	281	157,567	1,012	155,509	999
Females	367,500 (49.2)	93,875	623	88,254	586	347,695	2,304	343,734	2,279
Missing data	0 (0)	153	–	216	–	146	–	204	–
Total	747,179 (100.0)	136,953	–	132,101	–	505,408	–	499,447	–
Ethnicity									
Males									
White	253,349 (66.7)	32,328	311	33,272	321	141,214	1,359	138,294	1,331
Black	25,152 (6.6)	586	57	756	73	740	72	933	90
Asian	68,944 (18.2)	2,575	91	2,399	85	5,105	180	5,026	177
Mixed	20,300 (5.3)	1,036	124	1,121	135	1,092	131	1,286	154
Other	11,933 (3.1)	1,692	346	1,596	326	2,320	474	2,413	493
Missing data	0 (0)	4,708	–	4,487	–	7,096	–	7,557	–
Females									
White	245,526 (66.8)	73,822	733	69,294	733	312,515	3,101	307,740	3,054
Black	24,096 (6.6)	1,486	150	1,680	150	2,179	220	2,440	246
Asian	66,860 (18.2)	5,072	185	4,599	185	9,909	360	9,758	355
Mixed	20,220 (5.5)	2,439	294	2,620	294	4,341	522	4,572	550
Other	10,798 (2.9)	2,928	661	2,880	661	4,874	1,097	5,095	1,147
Missing data	0 (0)	8,128	–	7,181	–	13,877	–	14,129	–
Age group									
Males									
6 to 18	260,319 (68.6)	10,407	98	7,307	69	23,100	216	17,243	162
19 to 24	119,360 (31.4)	32,518	665	36,324	743	134,467	2,746	138,266	2,824
Missing data	0 (0.0)	0	–	0		0	–	0	–
Females									
6 to 18	247,049 (67.2)	24,434	242	25,436	251	43,041	425	39,159	386
19 to 24	120,451 (32.8)	69,441	1,405	106,665	2,160	304,654	6,160	304,575	6,159
Missing data	0 (0.0)	0	–	0	–	0	–	0	–
IMD^a^									
Males									
1	170,531 (44.2)	18,654	267	19,817	284	85,603	1,223	83,501	1,193
2	75,418 (19.6)	9,996	323	10,541	341	33,795	1,092	34,140	1,104
3	44,012 (11.4)	5,487	304	5,095	283	15,475	858	15,341	851
4	45,710 (11.9)	4,752	254	4,480	239	12,431	663	12,459	665
5	39,998 (10.4)	3,952	241	3,599	220	10,215	623	10,018	611
Missing data	9,993 (2.6)	84	–	99	–	48	–	194	–
Females									
1	157,562 (43.6)	39,409	610	39,460	611	165,908	2,565	165,828	2,564
2	72,166 (20.0)	23,534	795	22,900	773	84,303	2,844	84,850	2,863
3	42,487 (11.8)	12,105	695	10,791	620	40,399	2,317	39,435	2,262
4	42,281 (11.7)	10,223	590	8,208	474	32,222	1,857	30,514	1,759
5	37,262 (10.3)	8,420	551	6,739	441	24,717	1,615	22,971	1,502
Missing data	9,759 (2.7)	184	–	156	–	146	–	136	–

^a^ Neighbourhood‐level Index of Multiple Deprivation (IMD): 1 = Most deprived, IMD 5 = Least deprived.

### Temporal trends before and during the pandemic

**Figure 1:**
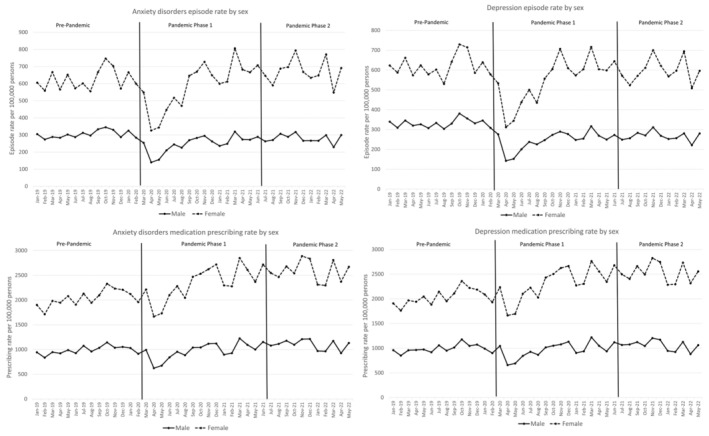
Monthly sex‐specific episode rates for anxiety disorders and depression diagnoses and for anxiolytic and antidepressant prescribing

There was a marked fall in episode rates of anxiety disorders and depression at the start of the COVID‐19 pandemic during the spring of 2020. Figure 1 shows these monthly counts for males and females (divided by the population denominator) as rates per 100,000 persons. By July 2021, rates rose to a similar level to that seen pre‐pandemic. Anxiolytics and antidepressant prescribing rates fell slightly in April and May 2020, but then a gradual increase (particularly noted in females) was observed.

Table 2 Shows the sex‐specific rate ratios (and 95% confidence intervals) for both phases of the pandemic compared to the pre‐pandemic. Rates of anxiety and depression were lower for Pandemic Phase 1, and generally, Pandemic Phase 2 rates were also marginally lower when compared to pre‐pandemic levels. During both Pandemic Phases, recorded episode rates for common mental illness fell more sharply in males than in females. In Pandemic Phase 1, prescribing rates in males were similar to those observed pre‐pandemic, whereas for females, increases in prescribing rates were observed. In Pandemic Phase 2, rates increased for both males and females rising above those seen pre‐pandemic. These increased prescribing rates were significantly higher in females than it was for males (relative percentage increases vs. males, anxiety disorders: 14%, 95% CI 3%–27%; depression: 16%, 95% CI 6%–28%).

**Table 2:** Temporal trends in rate ratios (vs. the pre‐pandemic period) stratified by sex – all episodes for anxiety disorders and depression and medication prescribed for these conditions (anxiolytics and/or antidepressants)

All episodes	Anxiety disorders		Depression	
	Pandemic phase 1	Pandemic phase 2	Pandemic phase 1	Pandemic phase 2
Males	0.82 (0.75–0.90)	0.91 (0.82–1.04)	0.74 (0.68–0.81)	0.80 (0.73–0.88)
Females	0.94 (0.85–1.04)	1.07 (0.96–1.19)	0.89 (0.82–0.97)	0.96 (0.87–1.06)

Medication	Prescribing for anxiety disorders		Prescribing for depression	
	Pandemic phase 1	Pandemic phase 2	Pandemic phase 1	Pandemic phase 2
Males	0.98 (0.92–1.05)	1.10 (1.02–1.18)	0.97 (0.91–1.04)	1.06 (0.99–1.13)
Females	1.14 (1.07–1.22)	1.25 (1.17–1.34)	1.13 (1.07–1.21)	1.23 (1.15–1.31)

### Sex‐specific temporal trends

**Figure 2:**
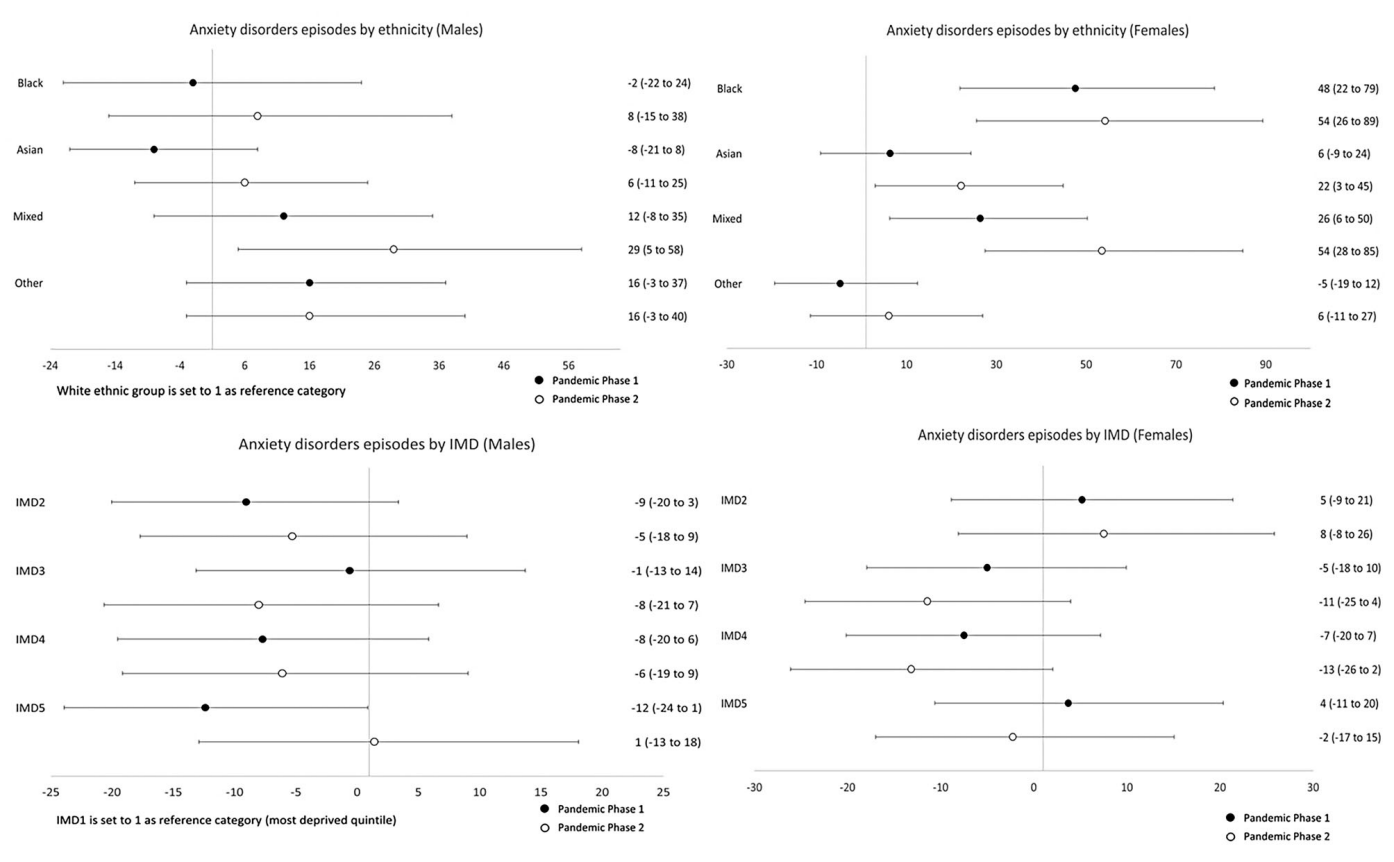
Relative percentage increases or decreases in rate ratios for minority ethnic groups (vs. White CYP) and for IMD quintiles 2–5 (vs. the most deprived quintile, IMD1) during Pandemic Phases 1 and 2: Anxiety disorders

**Figure 3:**
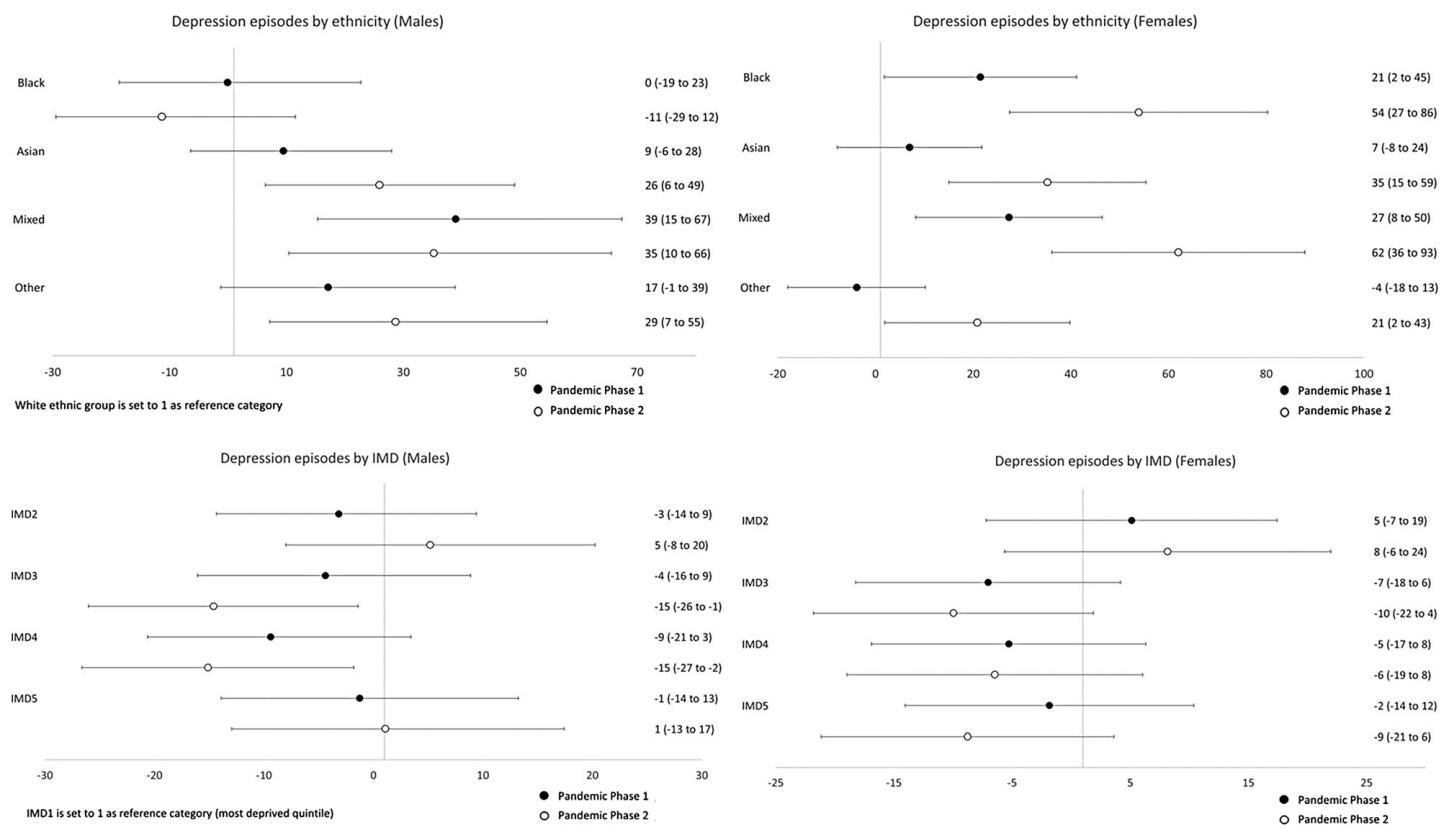
Relative percentage increases or decreases in rate ratios for minority ethnic groups (vs. White CYP) and for IMD quintiles 2–5 (vs. the most deprived quintile, IMD1) during Pandemic Phases 1 and 2: Depression

**Figure 4:**
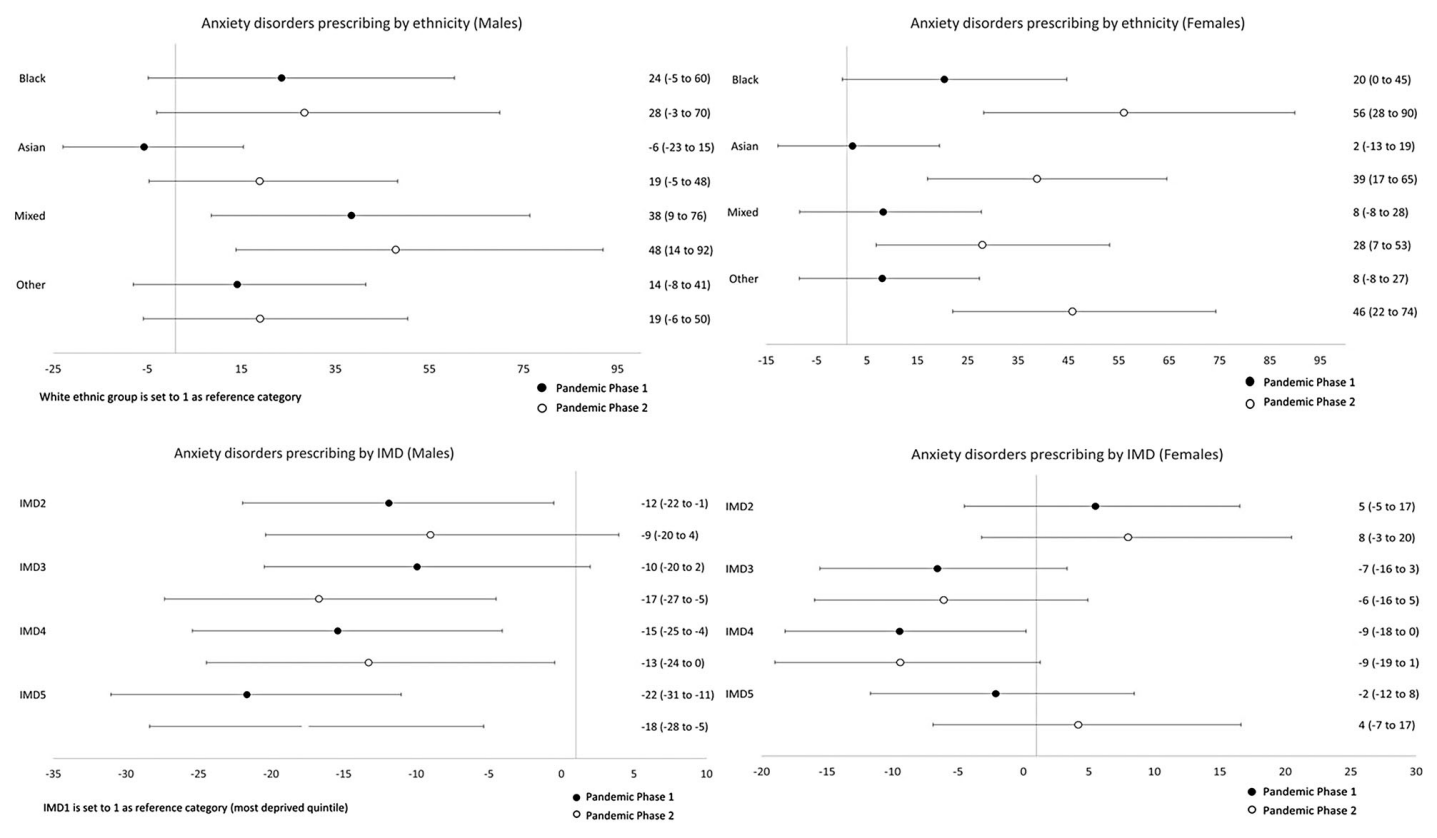
Relative percentage increases or decreases in rate ratios for minority ethnic groups (vs. White CYP) and for IMD quintiles 2–5 (vs. the most deprived quintile, IMD1) during Pandemic Phases 1 and 2: Prescribing of anxiolytics and/or antidepressants for anxiety disorders

**Figure 5:**
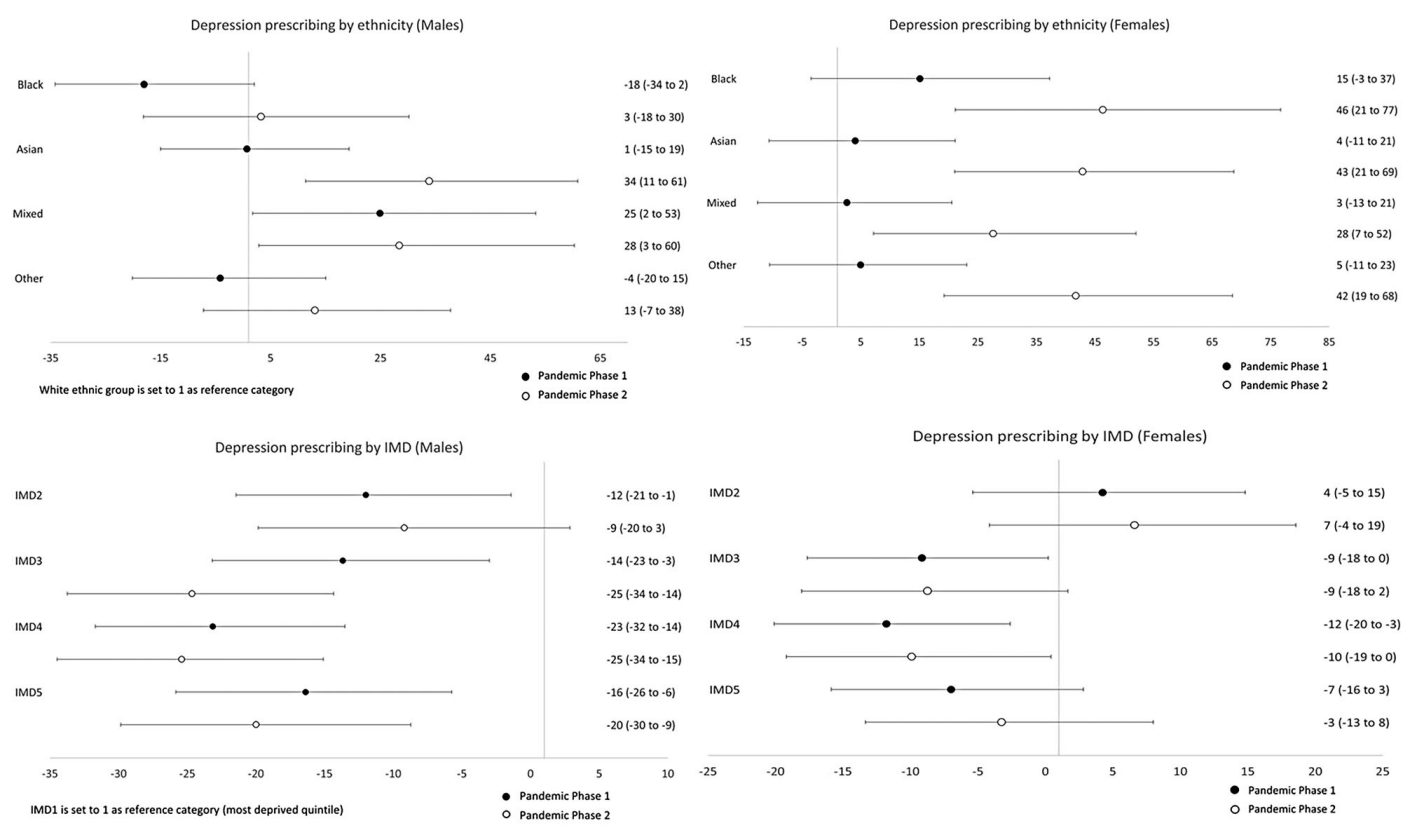
Relative percentage increases or decreases in rate ratios for minority ethnic groups (vs. White CYP) and for IMD quintiles 2–5 (vs. the most deprived quintile, IMD1) during Pandemic Phases 1 and 2: Prescribing of anxiolytics and/or antidepressants for depression

Table 3 presents sex‐specific rate ratios for ethnicity and IMD, comparing rates during Pandemic Phases 1 and 2 with pre‐pandemic levels. To examine whether these rate ratios differ by ethnicity and IMD, we analysed the interaction between ethnicity/IMD and time period. This tells us whether the rate of change was different between two ethnic or IMD groups. These analyses are illustrated in Figures 2, 3, 4, 5 where the ratios are expressed as a percentage increase or decrease compared to the reference categories (White ethnic group and the most deprived quintile (IMD1) were set to 1). Rates per 100,000 persons for all three study time periods are illustrated graphically in Figures S1–S4.

**Table 3:** Temporal trends in sex‐specific rate ratios (vs. the pre‐pandemic period) by ethnic group and IMD quintile – all episodes for anxiety disorders and depression and medication prescribed for these conditions (anxiolytics and/or antidepressants)

	Episodes				Medication			
	Anxiety disorders		Depression		Prescribing for anxiety disorders		Prescribing for depression	
	Pandemic phase 1	Pandemic phase 2	Pandemic phase 1	Pandemic phase 2	Pandemic Phase 1	Pandemic Phase 2	Pandemic Phase 1	Pandemic Phase 2
Ethnicity								
Males								
White	0.81 (0.74–0.89)	0.89 (0.81–0.99)	0.73 (0.66–0.80)	0.78 (0.71–0.86)	0.97 (0.84–1.11)	1.08 (0.93–1.25)	0.96 (0.86–1.08)	1.03 (0.92–1.17)
Black	0.78 (0.64–0.96	0.97 (0.79–1.19)	0.71 (0.58–0.87)	0.69 (0.56–0.86)	1.16 (0.89–1.50)	1.41 (1.07–1.86)	0.79 (0.63–0.99)	1.07 (0.84–1.35)
Asian	0.73 (0.65–0.83)	0.94 (0.83–1.07)	0.79 (0.69–0.89)	0.99 (0.87–1.14)	0.90 (0.77–1.06)	1.29 (1.09–1.53)	0.95 (0.83–1.09)	1.39 (1.20–1.60)
Mixed	0.91 (0.78–1.07)	1.18 (1.01–1.39)	1.04 (0.89–1.20)	1.03 (0.88–1.21)	1.38 (1.04–1.84)	1.67 (1.23–2.27)	1.25 (0.98–1.59)	1.33 (1.02–1.72)
Other	0.96 (0.83–1.11)	1.03 (0.89–1.20)	0.85 (0.75–0.96)	1.01 (0.88–1.15)	1.11 (0.98–1.25)	1.27 (1.12–1.44)	0.92 (0.83–1.02)	1.17 (1.05–1.30)
Females								
White	0.93 (0.84–1.03)	1.02 (0.92–1.14)	0.88 (0.80–0.97)	0.91 (0.82–1.01)	1.14 (1.02–1.26)	1.22 (1.09–1.37)	1.13 (1.02–1.26)	1.20 (1.07–1.34)
Black	1.35 (1.15–1.59)	1.57 (1.32–1.86)	1.06 (0.94–1.21)	1.38 (1.21–1.58)	1.41 (1.18–1.68)	1.90 (1.58–2.29)	1.31 (1.13–1.53)	1.74 (1.48–2.05)
Asian	0.98 (0.87–1.09)	1.26 (1.11–1.42)	0.94 (0.82–1.08)	1.23 (1.06–1.43)	1.16 (1.03–1.30)	1.70 (1.50–1.93)	1.18 (1.02–1.37)	1.74 (1.48–2.04)
Mixed	1.18 (1.00–1.38)	1.60 (1.35–1.89)	1.11 (0.97–1.28)	1.47 (1.27–1.69)	1.21 (1.06–1.38)	1.58 (1.38–1.81)	1.15 (1.05–1.27)	1.53 (1.39–1.68)
Other	0.89 (0.80–0.99)	1.07 (0.96–1.20)	0.84 (0.75–0.95)	1.11 (0.99–1.25)	1.23 (1.09–1.38)	1.80 (1.59–2.03)	1.19 (1.05–1.36)	1.71 (1.49–1.96)
IMD^a^								
Males								
IMD1	0.86 (0.78–0.93)	0.94 (0.85–1.03)	0.76 (0.70–0.83)	0.81 (0.74–0.89)	1.05 (0.96–1.14)	1.16 (1.06–1.28)	1.05 (0.97–1.14)	1.15 (1.05–1.25)
IMD2	0.78 (0.71–0.86)	0.89 (0.81–0.99)	0.73 (0.67–0.80)	0.85 (0.78–0.94)	0.92 (0.86–0.98)	1.05 (0.98–1.13)	0.92 (0.87–0.98)	1.04 (0.97–1.11)
IMD3	0.85 (0.78–0.94)	0.85 (0.77–0.94)	0.72 (0.67–0.79)	0.69 (0.63–0.76)	0.95 (0.86–1.04)	0.97 (0.87–1.07)	0.91 (0.83–0.99)	0.86 (0.78–0.94)
IMD4	0.80 (0.73–0.87)	0.88 (0.80–0.97)	0.69 (0.62–0.76)	1.12 (1.07–1.18)	0.88 (0.79–0.98)	1.01 (0.89–1.13)	0.80 (0.72–0.88)	0.86 (0.77–0.96)
IMD5	0.73 (0.66–0.82)	0.95 (0.85–1.07)	0.75 (0.67–0.83)	0.82 (0.73–0.92)	0.82 (0.74–0.90)	0.96 (0.86–1.06)	0.88 (0.81–0.97)	0.93 (0.84–1.02)
Females								
IMD1	0.95 (0.86–1.05)	1.08 (0.97–1.21)	0.90 (0.82–0.98)	0.97 (0.88–1.06)	1.15 (1.07–1.23)	1.25 (1.15–1.35)	1.16 (1.08–1.24)	1.24 (1.15–134)
IMD2	1.00 (0.90–1.12)	1.17 (1.04–1.31)	0.94 (0.85–1.03)	1.05 (0.95–1.16)	1.22 (1.14–1.30)	1.35 (1.25–1.45)	1.21 (1.13–1.29)	1.32 (1.23–1.42)
IMD3	0.91 (0.82–1.02)	0.96 (0.85–1.08)	0.84 (0.76–0.92)	0.87 (0.79–0.96)	1.07 (1.00–1.15)	1.17 (1.09–1.26)	1.05 (0.99–1.12)	1.13 (1.06–1.21)
IMD4	0.87 (0.78–0.97)	0.94 (0.82–1.06)	0.85 (0.77–0.93)	0.91 (0.82–1.00)	1.04 (0.96–1.13)	1.13 (1.04–1.23)	1.02 (0.94–1.10)	1.12 (1.02–1.22)
IMD5	0.98 (0.88–1.10)	1.06 (0.94–1.19)	0.88 (0.81–0.97)	0.88 (0.80–0.97)	1.12 (1.03–1.23)	1.30 (1.18–1.43)	1.07 (0.99–1.17)	1.20 (1.09–1.31)

^a^ Neighbourhood‐level Index of Multiple Deprivation (IMD): 1 = Most deprived, IMD 5 = Least deprived.

### Temporal trends by ethnicity

For males, generally, rates of anxiety disorders and depression for Pandemic Phase 1 were lower compared to the pre‐pandemic rates and then rose during Pandemic Phase 2, returning to a similar level to that observed pre‐pandemic. There was one exception to this general pattern; the rate of depression in Black males continued to decrease with a rate ratio of 0.69 (95% CI 0.56–0.86) in Pandemic Phase 2. For females, some notable differences between the ethnic groups were observed. For White and Other ethnic groups, there was an initial reduction in rates of both anxiety disorders and depression followed by an increase to levels similar to those seen pre‐pandemic. In contrast, for females within the Black and Mixed ethnic groups, there was an increase in rates of both anxiety disorders and depression. In Pandemic Phase 2, rates for anxiety disorders increased by 57% (1.57, 95% CI 1.32–1.86) in Black females and 60% (1.60, 95% CI 1.35–1.89) in females of mixed ethnicity. In addition, there was a significant increase in the rate of change when compared to those of White ethnicity. During Pandemic Phase 2, rates for anxiety disorders increased by approximately a half for Black (relative percentage increase vs. White: 54%,95% CI 26%–89%) and Mixed (54%, 95% CI 28%–85%) ethnic groups. Similar increases were seen in rates for depression, with a relative percentage increase in rates in Black (54%, 95% CI 27%–86%) and Mixed (62%, 95% CI 36%–93%) females (compared to White females) during the second phase of the Pandemic.

In males, Pandemic Phase 2 anxiolytic/antidepressant prescribing rates were higher than pre‐pandemic levels, although a decrease in rates was noted in some ethnic groups in Pandemic Phase 1. A marked increase in prescribing for anxiety disorders was observed in males of Black and Mixed ethnicity, the latter increasing by 67% (95% CI 23%–227%) in Pandemic Phase 2. This increase in rate was 48% (95% CI 14%–92%) higher than the rate for the White ethnic group. For females, prescribing rates for both anxiety disorders and depression increased for all ethnic groups during both phases of the Pandemic. Compared to pre‐pandemic rates, the prescribing rate for anxiety disorders seen in Black females almost doubled during Pandemic Phase 2 (RR 1.90, 95% CI 1.58–2.29) and increased rates of 70% (95% CI 50–93%) were seen in females within Asian and 80% (95% CI 59%–203%) for Other ethnic groups. Increased prescribing rates were also observed in these ethnic groups for episodes of depression. Increased rates within these ethnic groups (and also in those of Mixed ethnicity) were also shown to be significantly different than that observed in White females (Figures 4 and 5).

### Temporal trends by indices of multiple deprivation quintile

In Table 3, Figures S1–S4 and Figures 2, 3, 4, 5, the results for IMD have been shown alongside those for ethnicity, as described above. Overall, analysis by IMD showed a decrease in all quintiles in rates of anxiety disorders and depression in both phases of the pandemic compared to pre‐pandemic rates. In females, there was a gradual increase in the prescribing rate of anxiolytics and antidepressants during Pandemic Phases 1 and 2 for both diagnoses. A different pattern was observed in males showing a decrease in prescribing rates in Pandemic Phase 1 compared to pre‐pandemic rates.

The analysis further stratified by both ethnicity and IMD would likely be informative. However, it was not feasible to conduct these additional analyses because such granular stratification would generate multiple small numerator counts. As persons of White ethnicity constituted 67% of the study population, we conducted analysis by IMD quintile separately for the White ethnic group and all other ethnicities combined. These rates (per 100,000 persons) are presented in the supporting information (Figures S5–S8). This further stratification revealed an ethnic disparity in the overall rate of the most deprived quintile relative to the other four IMD quintiles. For the White ethnic group, the most deprived quintile shows some of the highest rates of common mental disorders and related prescribing. However, for all other ethnicities combined, in the most deprived quintile, rates of anxiety disorders and depression were relatively lower than for most other IMD quintiles. In addition, compared to the White ethnic group, those of all other ethnicities combined showed increased rates over time.

## Discussion

### Summary of main findings

Rates of anxiety disorders and depression were highest in females, older CYP and those of White and Other ethnic groups. Overall, during the first phase of the Pandemic, there was a fall in the recording of anxiety disorders and depression observed in all demographic subgroups. Increased rates for these common mental disorders were observed during the second phase of the Pandemic, with rates in most subgroups returning to similar levels as those recorded pre‐pandemic. In this later phase of the study observation period, there were, however, some notable deviations from these general findings. Compared to pre‐pandemic levels, rates in females of Black and Mixed ethnicity rose significantly, at a greater rate than those of White ethnicity. Conversely, there was no notable reduction in anxiolytics and antidepressants prescribing during the first phase of the Pandemic and the prescribing rate increased significantly during the second phase. Increased prescribing was particularly evident in ethnic minorities, with significant differences in rates compared to the White ethnic group. This was particularly marked in Black females; prescribing rates for anxiety disorders among these patients almost doubled during the second phase of the pandemic. This increase in rate was 56% higher than the increased rate shown in White female patients. There was, however, little significant difference when comparing the rates of change between the five neighbourhood‐level deprivation quintiles.

### Comparison of findings with other published evidence

Consistent with other published literature (Carr et al., 2021; Steeg et al., 2021; Williams et al., 2020), there was a decrease in rates of anxiety disorders and depression recorded in CYP in the early stages of the COVID‐19 Pandemic. Data were extracted from the GMCR, which is based on clinical codes that have been entered into the patient's primary care electronic health record. As such, these results may not only reflect real changes in the prevalence of the condition but also in the likelihood of an individual presenting to healthcare services and that information being recorded. Patients' access to care changed fundamentally due to COVID‐19, with consultation rates reported as decreasing by up to 33% (Carr et al., 2021; Clarke, Pariza, & Wolters, 2020; Steeg et al., 2021; Trafford et al., 2023; Williams et al., 2020). A study conducted by Carr et al. used electronic health records from the UK Clinical Practice Research Datalink (CPRD) to examine the effects of the pandemic on mental illness and self‐harm. Rates of anxiety disorders and depression diagnosis among patients aged 10–17 years in the first 6 months of the pandemic were approximately half the rates observed pre‐pandemic (Carr et al., 2021). By examining the Salford Integrated Record dataset (in GM), Williams et al also found a reduction of 50% in diagnosed common mental health problems compared to pre‐pandemic rates. (Williams et al., 2020). In Pandemic Phase 1 of our study, the rate of recognised anxiety disorders reduced by 18% for males and 6% for females, and for depression, a reduction of 26% (males) and 11% (females) was observed. The reduction in the Phase 1 of our study was less than in these others described as it was over a longer period (16 months) including later months when rates started to rise. Conversely, research based on self‐reports describes decreases in mental well‐being during this early COVID‐19 period. A survey based on the General Health Questionnaire (GHQ‐12) reported psychological distress rose from 19% in 2018–2019 to 27% in April 2020 (Pierce et al., 2020) and another evaluating psychological well‐being (using the Warwick‐Edinburgh Mental Well‐being Scale) found psychological distress in 50% of the population in June/July 2020, a three‐to‐four‐fold increase on pre‐pandemic prevalence (Gray et al., 2020). Similar findings were reported with a significant rise in depressive symptoms measured using the Revised Child Anxiety and Depression Scale (RCADS) (Bignardi et al., 2021).

Our results show that this early pandemic decrease in rates of anxiety disorders and depression was not reflected in the rates for anxiolytic/antidepressant prescribing. Other studies examining temporal trends in prescribing reported a decrease, but to a much lesser extent than was observed for mental health diagnoses (Carr et al., 2021; Williams et al., 2020). Both these studies state that electronic repeat prescribing is likely to have mitigated the larger decreases observed for anxiety disorders and depression (Carr et al., 2021) as the recording of prescriptions issued is an automated process and likely to represent more accurate changes in longitudinal tends (Williams et al., 2020). We could, therefore, assume that the consistent rise in anxiolytic/antidepressant prescribing shown here may reflect a truer picture of the change in the incidence of these primary care recorded common mental ill‐health diagnoses.

Across the whole 41‐month study period, rates of all four outcome measures were highest in those of White and Other ethnicities and lowest in the Black ethnic group. Evidence in the published literature is conflicting with studies examining racial inequality in mental health, most frequently finding prevalence highest in those of Black ethnicity (Smith, Bhui, & Cipriani, 2020). A survey carried out by NHS Digital in 2014 reported prevalence rates of common mental disorders as White British‐17%, White Other‐14%, Black/Black British‐23%, Asian/Asian British‐18%, Mixed/Other‐20%. However, this survey also found that despite this higher prevalence, those of Black ethnicity had the lowest treatment rate for mental ill‐health of any ethnic group (6% compared to 13% in those of White British ethnicity; McManus, Bebbington, Jenkins, & Brugha, 2016). This was also shown in a study by Ahmad et al. in which analysis of survey data found that people of Black ethnicity had the highest prevalence of common mental disorders but the lowest odds of having received relevant treatment (Ahmad, McManus, Cooper, Hatch, & Das‐Munshi, 2022). In addition, a systematic review examining inequalities experienced by ethnic minority groups found that Black populations were less likely to access mental health support via traditional pathways and sought help elsewhere (e.g. in the community). This meant that when services were accessed, they were experiencing higher levels of mental distress and nearer crisis points than other ethnic groups (Devonport et al., 2022). It is difficult to know why rates observed in the Other ethnic group are similar to those of White ethnicity, that is whether it is an artefact of missing data, misclassification or a real phenomenon. Watkinson et al used GMCR data to examine ethnic inequalities in COVID‐19 vaccine uptake. The Office for National Statistics (ONS) classifies ethnicity into 20 different groups, whereas within the GMCR, the population is grouped into five categories. The authors suggested this lack of granularity could lead to an over‐classification into the Other ethnic group, and therefore, lead to inflated estimates in incidence/prevalence/prescribing (Watkinson, Williams, Gillibrand, Sanders, & Sutton, 2022). It is interesting to note that in the NHS Digital figures quoted above, the Mixed & Other group has the second highest prevalence. According to ONS, the Other ethnic group is made up of 33% ‘Arab’ and 67% ‘Any other ethnic group’ (Office for National Statistics, 2023). A Government survey carried out in 2018 reported results from a self‐reported anxiety survey. Individuals describing themselves as Arab had the highest average anxiety score. This may contribute to the high rates in the Other category described here (Office of National Statistics, 2019).

Given the difficulty in evaluating how much our study results are due to differences in health‐seeking behaviour or a true reflection of incidence/prevalence, the primary interest is in the change over time, particularly by ethnicity. The most notable findings were in the increases in rates of anxiety disorders and depression in ethnic minorities during Pandemic Phase 2 compared to pre‐pandemic levels, particularly in females. This trend is also shown in the prescribing rates for anxiolytics and antidepressants for these conditions. In addition, our results show a significant increase in the rate of change compared to those of White ethnicity. This pattern is particularly evident as regards prescribing for anxiety disorders among Black females. The prescribing rate almost doubled compared to pre‐pandemic levels, and this increased at a 56% higher rate than for the White ethnic group. This suggests that as time progresses, Black females are not only experiencing increased levels of mental ill‐health but also having increased contact with healthcare services. The review by Devenport et al suggests that affected individuals of Black ethnicity seek professional help at a higher threshold than people of other ethnicities. Therefore, the observed increase in rates in this group may be explained by an increase in the level of distress in individuals already experiencing symptoms but previously undiagnosed rather than (or in addition to) an increase in the number of individuals who experienced distress (Devonport et al., 2022). This may also explain the increased level of prescribing in these individuals, that is those who are more distressed are more likely to be treated with medication. Connolly (2010) reviewed published evidence of ethnical disparity in prescribing and concluded that in the UK, treatment using antidepressants appeared to be broadly equitable for minority ethnic groups. However, evidence in the UK for this complex issue is lacking and further investigation of the numerous reasons for these differences is urgently needed (Connolly, 2010). A study by Proto et al aimed to compare mental well‐being in April 2020 to pre‐pandemic levels using the UK Household Longitudinal Study. The authors found that rates of mental distress were highest for BAME (Black, Asian and minority ethnic) females during both survey periods, although the average increase was highest for all females (regardless of ethnicity) and BAME men (Proto & Quintana‐Domeque, 2021). Conversely, two systematic reviews examining rates of mental distress pre and ‘peri’ COVID found no evidence of any differential change by ethnicity (Patel et al., 2022; Schafer, Lieberman, Sever, & Joiner, 2022).

Evidence has shown that during the pandemic, people living within the most socially deprived neighbourhoods had greater increases in self‐reported mental ill‐health (Griggs, Horvat Davey, Howard, Pignatiello, & Duwadi, 2022; Kwong et al., 2021; Shevlin et al., 2020). However, they also had lower levels of primary care recorded mental illness, suggesting inequalities in access/help‐seeking behaviour (Carr et al., 2021). In our study, we did not find evidence of any marked disparity by neighbourhood‐level deprivation between the five IMD quintiles in the rate of change with time. However, throughout our study period, there were notable differences in the episode and prescribing rates between the IMD quintiles, particularly upon further stratification by ethnicity. It is known that ethnicity and deprivation are correlated. A Government report from 2019 stated that people from ethnic minorities were more likely to live in the 10% most deprived neighbourhoods compared to those of White ethnicity (Office for National Statistics, 2020). A King's Fund report (2021) found that individuals from some ethnic minority groups are more likely than those of White ethnicity to report poorer health but also have poorer experiences of using health services (Raleigh & Homles, 2021). The previously discussed challenges/barriers in accessing healthcare among ethnic minority groups may be more impactful among those living in more deprived areas. Analysis of a national cohort study found that although rates of adolescent mental health were highest in some ethnic minority groups (Black Caribbean, Mixed and Other Ethnic Group), these differences were largely attenuated when results were adjusted by household income (Ahmad, McManus, Bécares, Hatch, & Das‐Munshi, 2022).

Our study examines the association between the COVID‐19 pandemic and primary care activity related to anxiety and depressive disorders and suggests it may have had an important impact on the mental health of CYP. The temporal increases in rates of anxiety disorders and depression and associated prescribing in ethnic minorities suggest a disparity in access to primary care and subsequent referral to mental health services. If affected individuals of Black, Asian and Mixed ethnicity have a higher threshold in help‐seeking behaviour than White people, this could be an indication of worsened severity (in those with existing, as yet undiagnosed symptoms of distress) of mental distress as well as increased prevalence.

### Strengths and limitations

There have been no comparable studies examining ethnic disparities in CYPs' mental health during the COVID‐19 pandemic. Therefore, this study adds to the body of evidence regarding inequalities in mental well‐being, help‐seeking behaviour and health service utilisation. The use of GMCR data enabled us to examine information on 2.8 million people registered with GPs across the whole of GM. Given the relatively high level of deprivation in the conurbation our findings may not be generalisable nationally. In addition, due to the regional ‘Tiering System’ introduced in September 2020, the GM population spent longer under more restrictive lockdown measures than most other UK regions. A study examining the mental health of UK CYPs found that higher levels of lockdown severity were associated with increased depressive symptoms (Owens et al., 2022). The GMCR captures demographic data on ethnicity and deprivation enabling the examination of trends by these demographic groups. GMCR data is based on clinical codes entered into primary care systems resulting from GP‐patient contact or retrospectively following a hospital visit (Steeg et al., 2021). Therefore, a diagnosis will only be included if there has been interaction with healthcare services. Due to the availability of automated repeat prescription ordering systems, it is likely that the data on medication prescribed offers a more accurate picture of temporal trends. However, it is not known whether the COVID‐19 pandemic changed GPs' prescribing behaviour (and approval of prescription requests), with the possibility of an increase in prescribing acting as mitigation for the reduction in clinical contact. Prescribing data was based on the number of prescriptions issued for the outcomes of interest. As such, it is also not known whether there were any changes in prescription duration (i.e. the quantity prescribed) or any changes in dosage. GMCR data capturing clinical contact associated with anxiety disorders and depression is based on a list of relevant clinical codes. As such, we cannot determine whether the temporal changes we observed are changes in the relative rates of these conditions or in a change in the way GPs classify patients with an overlapping set of symptoms. We separated the 41‐month study period into three phases and compared Pandemic Phases 1 and 2 with the pre‐pandemic phase rather than to calculate monthly rates to optimise statistical precision. The allocation of months into these three phases may be considered subjective, although the authors pragmatically based this decision on notable changes in societal restrictions. GMCR data was not available prior to 2019, therefore, it was not possible to carry out optimal interrupted time‐series analyses as it was not possible to generate predicted frequencies during the pandemic based on prior trends. Although one of the main strengths of this study was the availability of ethnicity data, this was subject to some missing information (between 7.2% and 10.6% for our four outcomes of interest), which may have introduced bias. In the GMCR‐based study by Watkinson et al., the authors undertook an extensive evaluation of the demographic patterns of the missing ethnicity data and concluded that a large majority of those without this information were of White British ethnicity and that it did not substantially bias results (Watkinson et al., 2022).

## Conclusions

As highlighted elsewhere, a fundamental limitation of examining temporal trends using electronic health records is that we are unable to know to what extent the observed patterns reflect true increases in levels of distress and/or morbidity in the population as opposed to fluctuations in the propensity of affected individuals interacting with healthcare services. However, these analyses show rising frequencies in the diagnoses recorded and in the prescribing of associated medication. This indicates a greater demand being placed on an NHS that is struggling to meet demand post‐pandemic. We observed increasing numbers of non‐White CYP seeking support for mental health problems than was evident prior to March 2020. As such, healthcare care services in these areas will need to be adequately resourced to meet this rising demand, particularly within culturally diverse urban populations.

It is of utmost importance to identify the CYP most at risk of psychological ill‐health and to understand factors attributing to this increased risk. In addition, it is essential to identify barriers and facilitators to seeking and accessing help; early intervention may prevent deterioration in psychological well‐being. Further evidence on the impact of ethical disparity of access to primary care and treatment regarding prescribing and onward referral to therapeutic services is needed to prevent widening health inequality. Our findings add to the body of evidence on healthcare and educational provision regarding the potential impact of societal public health measures on the mental health of CYP, should future pandemics arise.

**Key points**

- The COVID‐19 pandemic has had a detrimental impact on the mental health of children and young people.
- Using GP electronic health records, we observed greater increases in the recorded diagnoses of anxiety disorders/depression and associated prescribing among adolescents within ethnic minority groups compared to those of White ethnicity.
- These results likely reflect service accessibility and patients' differential propensities to consult, as well as changing levels of psychopathology.
- It is essential to identify barriers and facilitators to seeking/accessing psychological support.

## Supporting information


**Table S1.** Incidence rate ratios for all episode/prescribing rates compared to reference categories for the four outcomes (anxiety disorders, depression, anxiolytics and/or antidepressants prescribed for these conditions) stratified by sex, age group, ethnic group and Index of Multiple Deprivation (IMD) quintile.
**Figure S1.** Temporal trends in sex‐specific anxiety disorders episode rates stratified by ethnic group and IMD quintile.
**Figure S2.** Temporal trends in sex‐specific depression episode rates stratified by ethnic group and IMD quintile.
**Figure S3.** Temporal trends in sex‐specific rates for prescribing of anxiolytics and/or antidepressants for anxiety disorders stratified by ethnic group and IMD quintile.
**Figure S4.** Temporal trends in sex‐specific rates for prescribing of anxiolytics and/or antidepressants for depression stratified by ethnic group and IMD quintile.
**Figure S5.** Temporal trends in sex‐specific anxiety disorders episode rates in White and All Other Ethnic Groups stratified by IMD quintile.
**Figure S6.** Temporal trends in sex‐specific depression episode rates in White and All Other Ethnic Groups stratified by IMD quintile.
**Figure S7.** Temporal trends in sex‐specific anxiolytic and/or antidepressant prescribing rates for anxiety disorders in White and All Other Ethnic Groups stratified by IMD quintile.
**Figure S8.** Temporal trends in sex‐specific anxiolytic and/or antidepressant prescribing rates for depression in White and All Other Ethnic Groups stratified by IMD quintile.

## Data Availability

Access to data are only available upon approval by the GMCR Research Governance Group.
