# Supplementary material for: Socio‐demographic variation in diagnosis of and prescribing for common mental illnesses among children and young people during the COVID‐19 pandemic: time series analysis of primary care electronic health records
Source: J Child Psychol Psychiatry. 2024 Jun 15;66(1):16–29. doi: 10.1111/jcpp.14026 (PMC11652419; doi:10.1111/jcpp.14026)
Supplement: Supplementary file 1 — Table S1. Incidence rate ratios for all episode/prescribing rates compared to reference categories for the four outcomes (anxiety disorders, depression, anxiolytics and/or antidepressants prescribed for these conditions) stratified by sex, age group, ethnic group and Index of Multiple Deprivation (IMD) quintile. Figure S1. Temporal trends in sex‐specific anxiety disorders episode rates stratified by ethnic group and IMD quintile. Figure S2. Temporal trends in sex‐specific depression episode rates stratified by ethnic group and IMD quintile. Figure S3. Temporal trends in sex‐specific rates for prescribing of anxiolytics and/or antidepressants for anxiety disorders stratified by ethnic group and IMD quintile. Figure S4. Temporal trends in sex‐specific rates for prescribing of anxiolytics and/or antidepressants for depression stratified by ethnic group and IMD quintile. Figure S5. Temporal trends in sex‐specific anxiety disorders episode rates in White and All Other Ethnic Groups stratified by IMD quintile. Figure S6. Temporal trends in sex‐specific depression episode rates in White and All Other Ethnic Groups stratified by IMD quintile. Figure S7. Temporal trends in sex‐specific anxiolytic and/or antidepressant prescribing rates for anxiety disorders in White and All Other Ethnic Groups stratified by IMD quintile. Figure S8. Temporal trends in sex‐specific anxiolytic and/or antidepressant prescribing rates for depression in White and All Other Ethnic Groups stratified by IMD quintile. [file JCPP-66-16-s001.docx]

**Supporting Information**

**Table S1.** Incidence rate ratios for all episode/prescribing rates compared to reference categories for the four outcomes (anxiety disorders, depression, anxiolytics and/or antidepressants prescribed for these conditions) stratified by sex, age group, ethnic group and Index of Multiple Deprivation (IMD) quintile.

|  | **Episodes** | | **Medication** | |
| --- | --- | --- | --- | --- |
|  | **Anxiety disorders** | **Depression** | **Anxiolytics** | **Antidepressants** |
| **Gender** |  |  |  |  |
| Males (Ref) | 1 | 1 | 1 | 1 |
| Females | 2.05 (1.86-2.25) | 1.87 (1.71-2.03) | 2.28 (2.14-2.42) | 2.28 (2.16-2.41) |
| **Ethnicity** |  |  |  |  |
| Males |  |  |  |  |
| White (Ref) | 1 | 1 | 1 | 1 |
| Black | 0.18 (0.15-0.21) | 0.24 (0.21-0.27) | 0.05 (0.05-0.06) | 0.07 (0.06-0.08) |
| Asian | 0.30 (0.27-0.33) | 0.24 (0.22-0.27) | 0.13 (0.12-0.15) | 0.13 (0.12-0.15) |
| Mixed | 0.36 (0.31-0.41) | 0.34 (0.30-0.40) | 0.10 (0.09-0.11) | 0.12 (0.10-0.13) |
| Other | 1.01 (0.89-1.14) | 0.90 (0.80-1.02) | 0.35 (0.31-0.39) | 0.37 (0.33-0.41) |
| Females |  |  |  |  |
| White (Ref) | 1 | 1 | 1 | 1 |
| Black | 0.15 (0.13-0.18) | 0.20 (0.18-0.23) | 0.07 (0.06-0.08) | 0.08 (0.07-0.09) |
| Asian | 0.23 (0.21-0.26) | 0.22 (0.20-0.24) | 0.12 (0.10-0.13) | 0.12 (0.10-0.13) |
| Mixed | 0.32 (0.28-0.36) | 0.36 (0.32-0.41) | 0.17 (0.15-0.19) | 0.18 (0.16-0.20) |
| Other | 0.90 (0.80-1.02) | 0.91 (0.81-1.02) | 0.35 (0.31-0.40) | 0.38 (0.34-0.42) |
| **Age Group** |  |  |  |  |
| Males |  |  |  |  |
| 6 to 18 (Ref) | 1 | 1 | 1 | 1 |
| 19 to 24 | 7.13 (6.37-7.99) | 10.76 (9.58-11.88) | 12.69 (11.69-13.80) | 17.47 (16.08-18.99) |
| Females |  |  |  |  |
| 6 to 18 (Ref) | 1 | 1 | 1 | 1 |
| 19 to 24 | 5.88 (5.15-6.72) | 8.34 (7.47-9.32) | 14.51 (13.30-15.84) | 15.94 (14.62-17.38) |
| **IMD*** |  |  |  |  |
| Males |  |  |  |  |
| IMD1 (Ref) | 1 | 1 | 1 | 1 |
| IMD2 | 1.27 (1.16-1.40) | 1.20 (1.10-1.31) | 0.89 (0.83-0.96) | 0.92 (0.86-0.99) |
| IMD3 | 1.17 (1.06-1.29) | 1.05 (0.96-1.16) | 0.70 (0.65-0.75) | 0.71 (0.67-0.76) |
| IMD4 | 1.00 (0.90-1.10) | 0.91 (0.83-0.99) | 0.54 (0.51-0.58) | 0.56 (0.52-0.60) |
| IMD 5 | 0.94 (0.85-1.04) | 0.78 (0.72-0.85) | 0.51 (0.47-0.55) | 0.51 (0.48-0.55) |
| Females |  |  |  |  |
| IMD1 (Ref) | 1 | 1 | 1 | 1 |
| IMD2 | 1.25 (1.13-1.39) | 1.21 (1.11-1.33) | 1.11 (1.04-1.18) | 1.12 (1.05-1.19) |
| IMD3 | 1.20 (1.08-1.34) | 1.07 (0.98-1.17) | 0.90 (0.85-0.97) | 0.88 (0.83-0.94) |
| IMD4 | 1.03 (0.93-1.15) | 0.80 (0.73-0.88) | 0.72 (0.68-0.77) | 0.69 (0.64-0.73) |
| IMD5 | 0.90 (0.80-0.99) | 0.74 (0.68-0.82) | 0.63 (0.59-0.67) | 0.59 (0.55-0.62) |

**Neighbourhood-level Index of Multiple Deprivation (IMD): 1=Most deprived, IMD 5=Least deprived*

**Figure S1.** Temporal trends in sex-specific anxiety disorders episode rates stratified by ethnic group and IMD quintile.


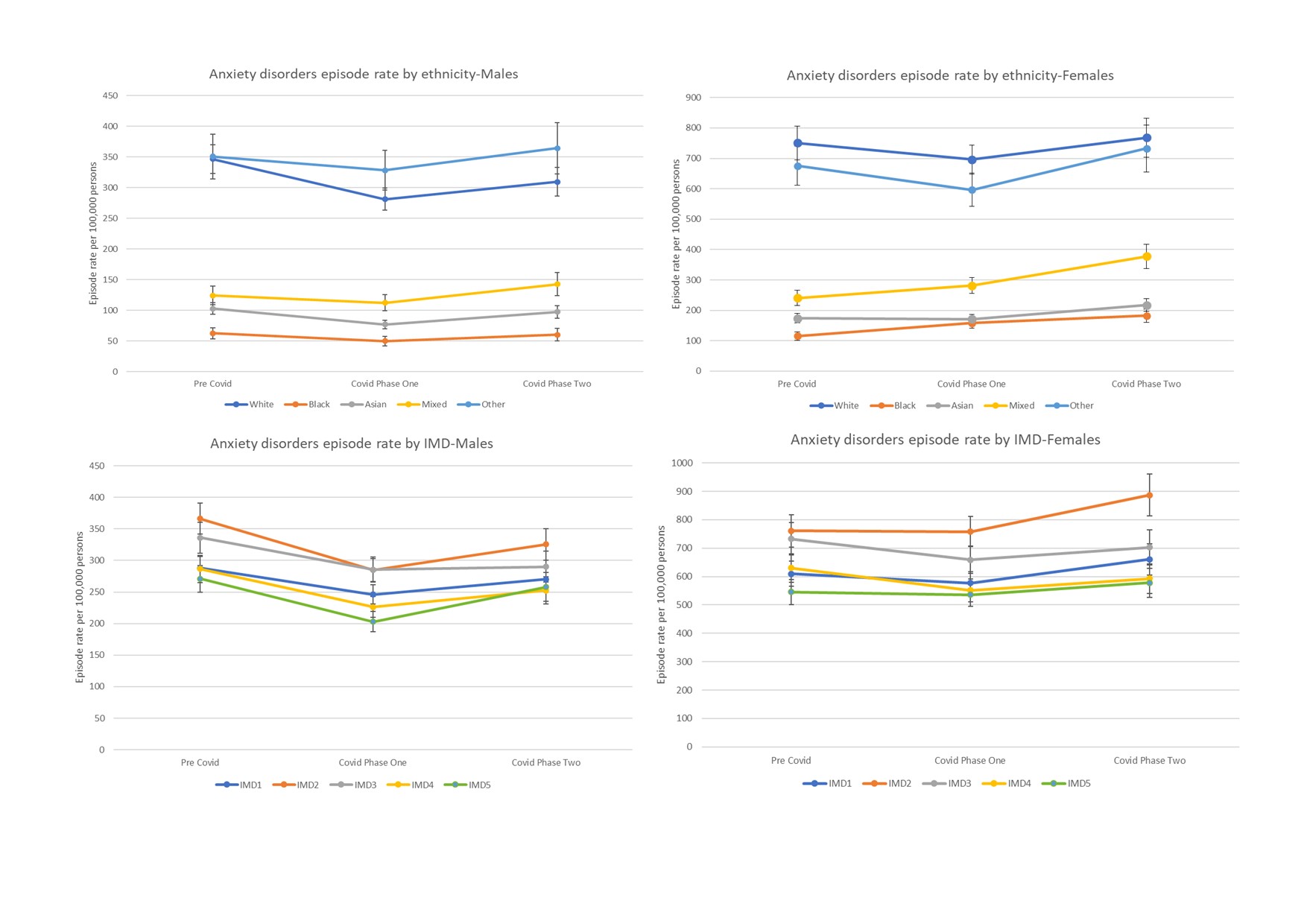


**Neighbourhood-level Index of Multiple Deprivation (IMD): 1=Most deprived, IMD 5=Least deprived*

**Figure S2.** Temporal trends in sex-specific depression episode rates stratified by ethnic group and IMD quintile.


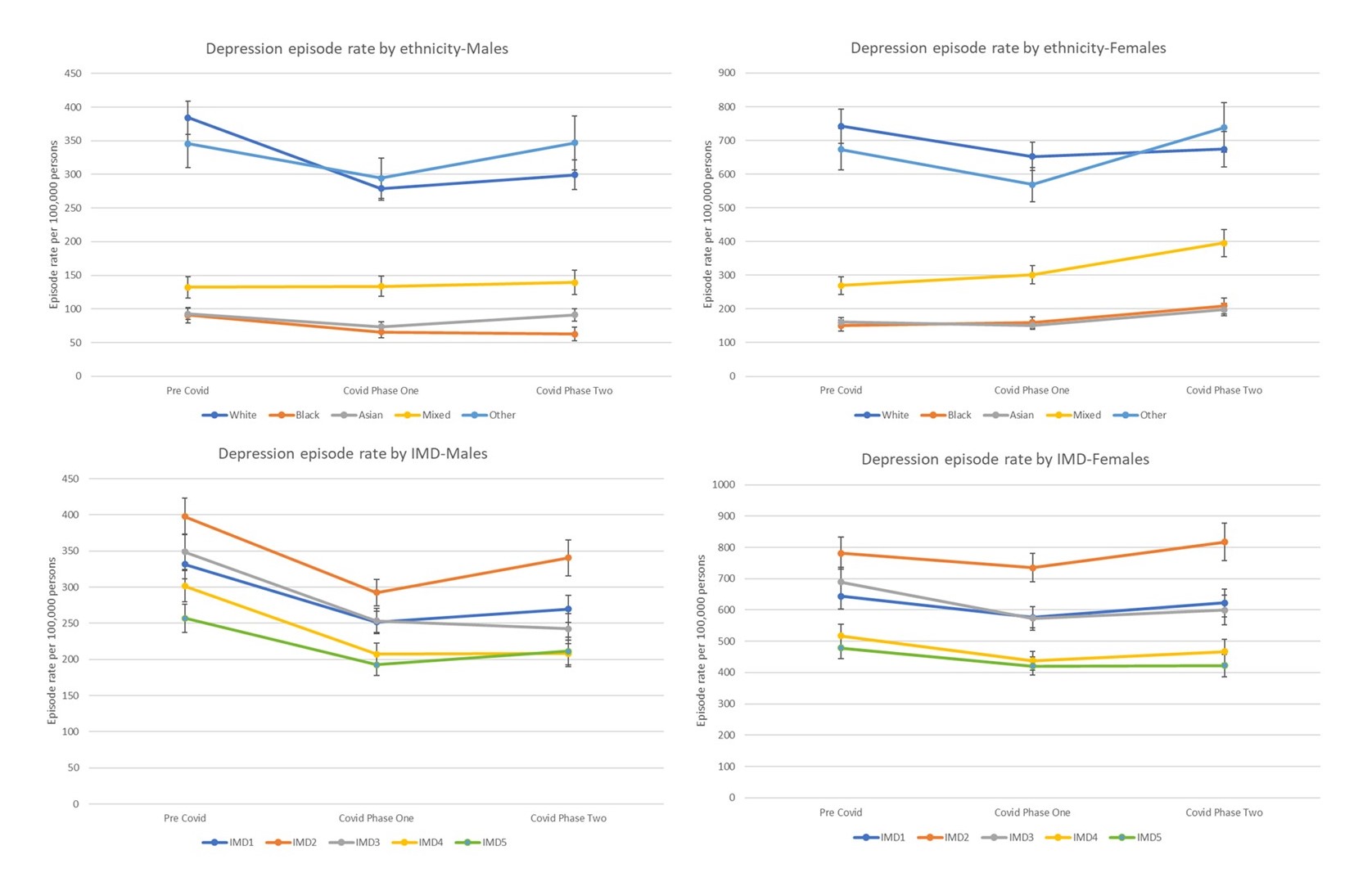


**Neighbourhood-level Index of Multiple Deprivation (IMD): 1=Most deprived, IMD 5=Least deprived*

**Figure S3.** Temporal trends in sex-specific rates for prescribing of anxiolytics and/or antidepressants for anxiety disorders stratified by ethnic group and IMD quintile.


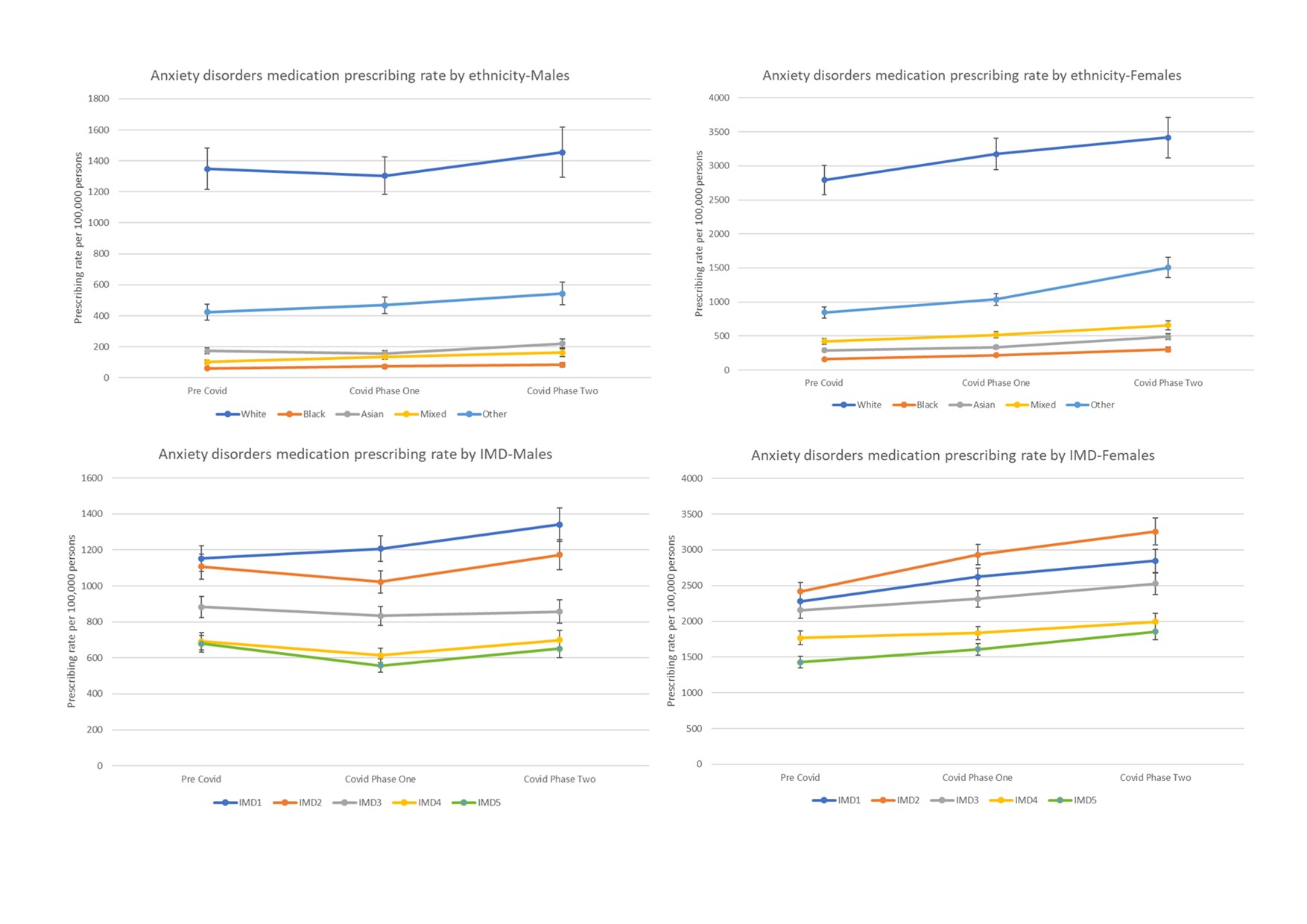


**Neighbourhood-level Index of Multiple Deprivation (IMD): 1=Most deprived, IMD 5=Least deprived*

**Figure S4.** Temporal trends in sex-specific rates for prescribing of anxiolytics and/or antidepressants for depression stratified by ethnic group and IMD quintile.


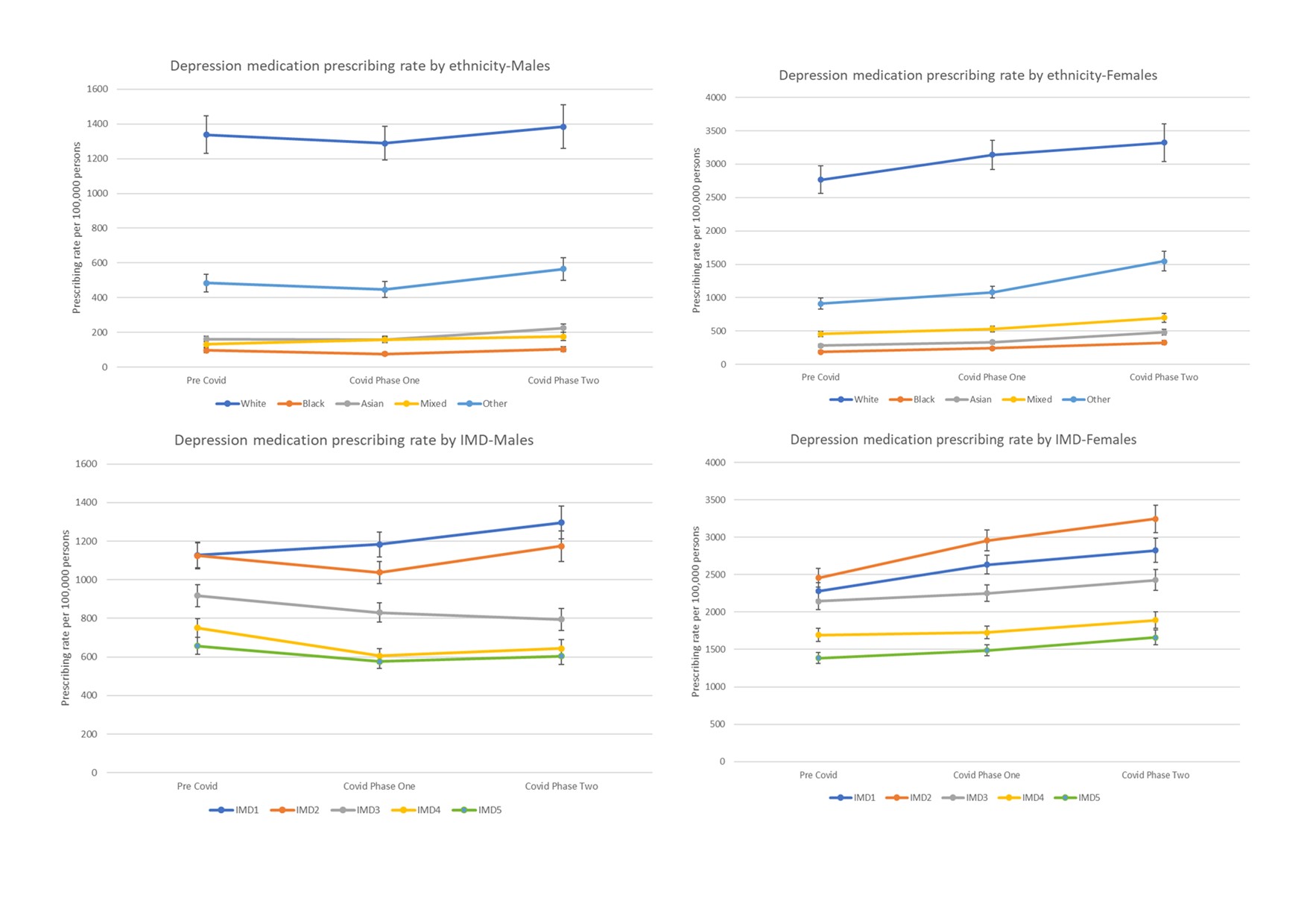


**Neighbourhood-level Index of Multiple Deprivation (IMD): 1=Most deprived, IMD 5=Least deprived*

**Figure S5.** Temporal trends in sex-specific anxiety disorders episode rates in White and All Other Ethnic Groups stratified by IMD quintile.


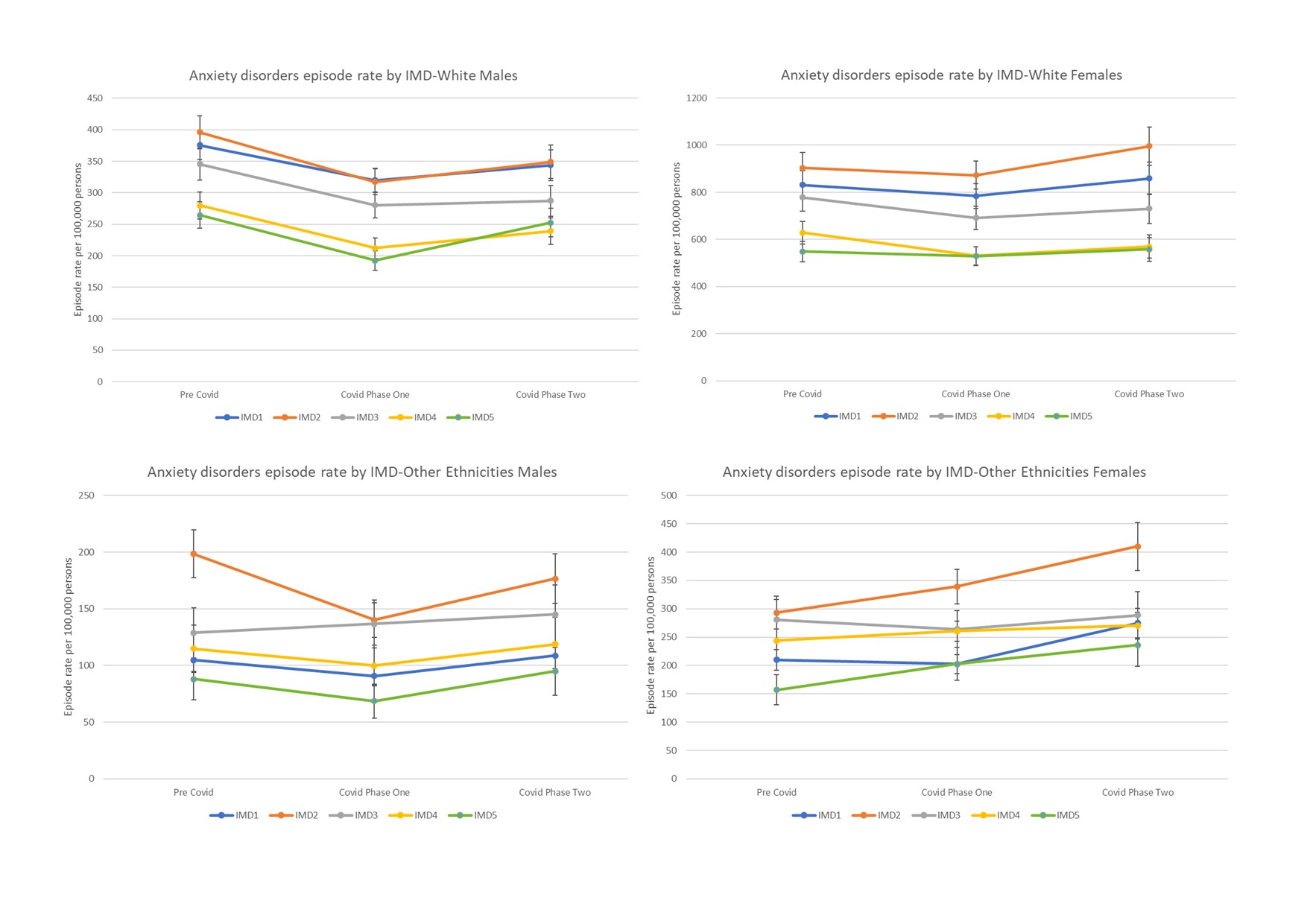


**Neighbourhood-level Index of Multiple Deprivation (IMD): 1=Most deprived, IMD 5=Least deprived*

**Figure S6.** Temporal trends in sex-specific depression episode rates in White and All Other Ethnic Groups stratified by IMD quintile.


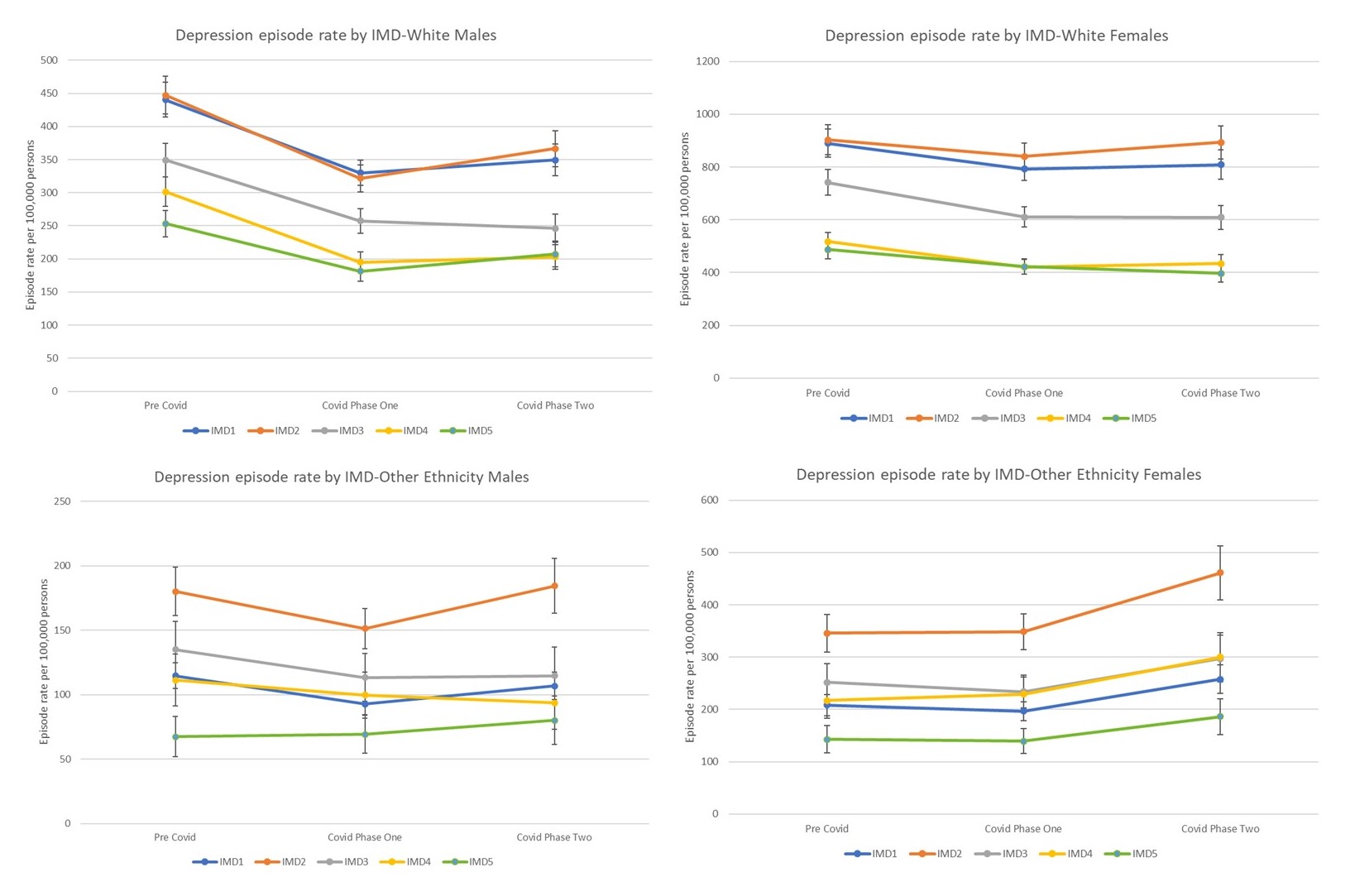


**Neighbourhood-level Index of Multiple Deprivation (IMD): 1=Most deprived, IMD 5=Least deprived*

**Figure S7.** Temporal trends in sex-specific anxiolytic and/or antidepressant prescribing rates for anxiety disorders in White and All Other Ethnic Groups stratified by IMD quintile.

**
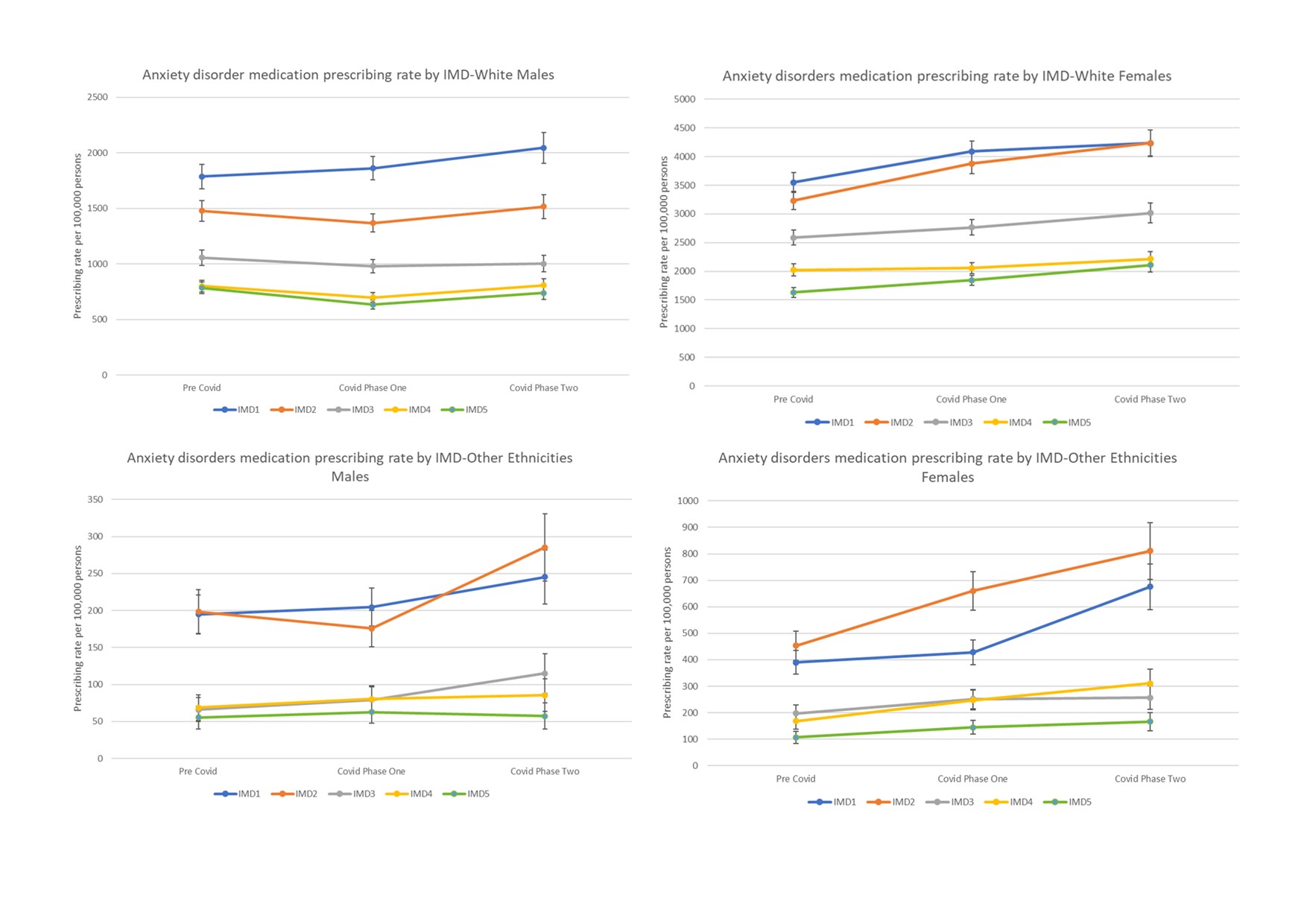
**

**Neighbourhood-level Index of Multiple Deprivation (IMD): 1=Most deprived, IMD 5=Least deprived*

**Figure S8.** Temporal trends in sex-specific anxiolytic and/or antidepressant prescribing rates for depression in White and All Other Ethnic Groups stratified by IMD quintile.


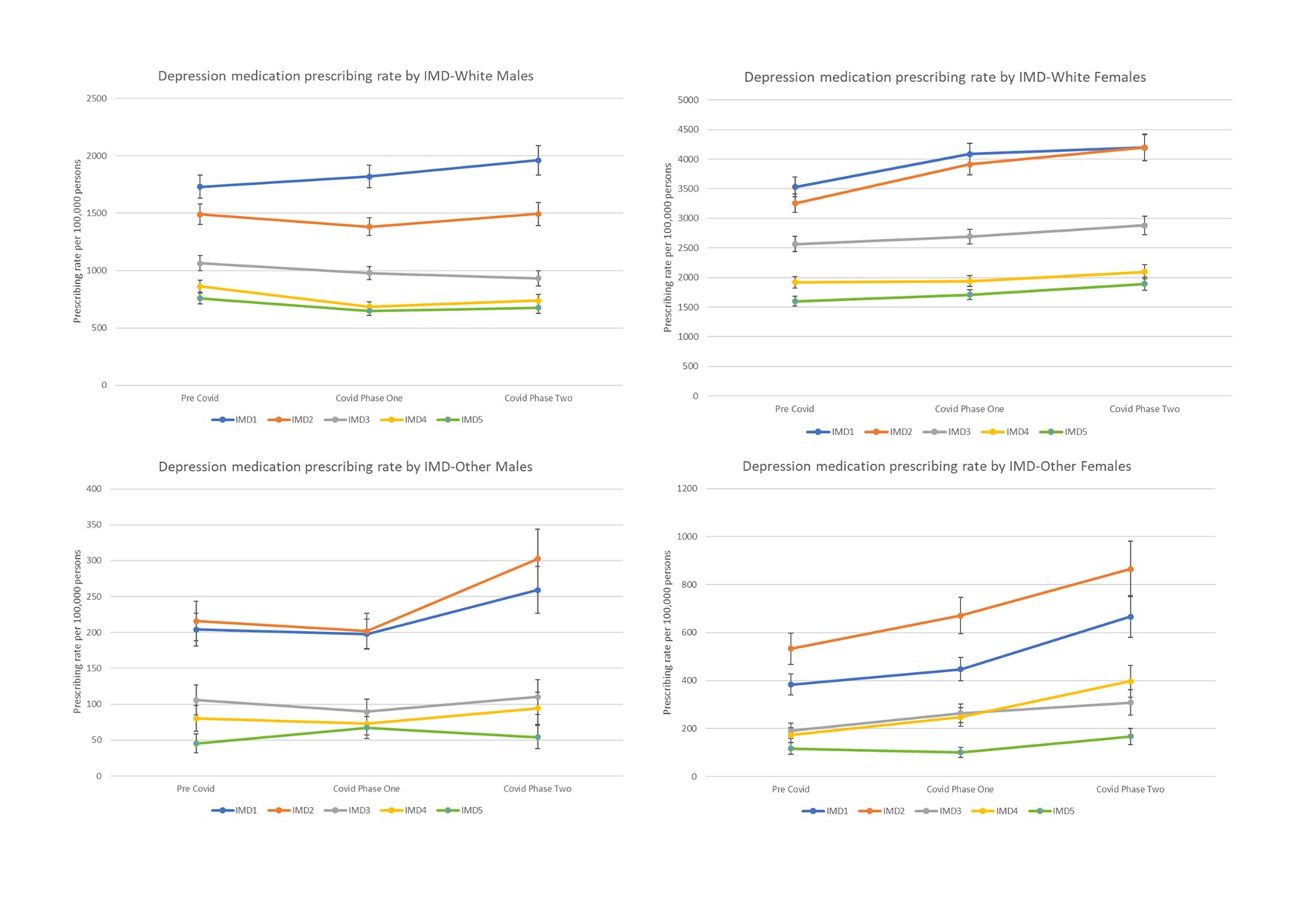


**Neighbourhood-level Index of Multiple Deprivation (IMD): 1=Most deprived, IMD 5=Least deprived*
